# MRI Imaging Characteristics of Glioblastoma with Concurrent Gain of Chromosomes 19 and 20

**DOI:** 10.3390/tomography7020021

**Published:** 2021-06-02

**Authors:** Taejin L. Min, Jason W. Allen, Jose E. Velazquez Vega, Stewart G. Neill, Brent D. Weinberg

**Affiliations:** 1 Department of Radiology and Imaging Sciences, Emory University School of Medicine, Emory University Hospital, Suite D112, 1364 Clifton Road NE, Atlanta, GA 30322, USA; tmin@emory.edu (T.L.M.); Jason.w.allen@emory.edu (J.W.A.); 2 Department of Pathology and Laboratory Medicine, Emory University School of Medicine, Emory University Hospital, Room H184, 1364 Clifton Road NE, Atlanta, GA 30322, USA; jose.enrique.velazquez.vega@emory.edu (J.E.V.V.); sgneill@emory.edu (S.G.N.)

**Keywords:** brain tumor, glioblastoma, imaging prognostication, radiomics, radiogenomics

## Abstract

Glioblastoma (GBM) is the most common and deadly primary brain tumor in adults. Some of the genetic variations identified thus far, such as *IDH* mutation and *MGMT* promotor methylation, have implications for survival and response to therapy. A recent analysis of long-term GBM survivors showed that concurrent gain of chromosomes 19 and 20 (19/20 co-gain) is a positive prognostic factor that is independent of *IDH* mutation status. In this study, we retrospectively identified 18 patients with 19/20 co-gain and compared their imaging features to a control cohort without 19/20 co-gain. Imaging features such as tumor location, size, pial invasion, and ependymal extension were examined manually. When compared without further genetic subclassification, both groups showed similar imaging features except for rates of pial invasion. When each group was subclassified by *MGMT* promotor methylation status however, the two groups showed different imaging features in a number of additional ways including tumor location, size, and ependymal extension. Our results indicate that different permutations of various genetic mutations that coexist in GBM may interact in unpredictable ways to affect imaging appearance, and that imaging prognostication may be better approached in the context of the global genomic profile rather than individual genetic alterations.

## 1. Introduction

Glioblastoma (GBM) is the most common malignant primary brain tumor in adults, with over 12,000 estimated new cases annually [1]. GBM also has the worst prognosis of all primary brain tumors, with median overall survival estimated at 15.6 months [2]. Current standard of care consisting of surgical resection, radiotherapy, and temozolomide was established in 2005, without significant advances made in survival rates since [2,3].

Although diagnosis of GBM is made on histopathologic grounds, there is increasing attention given to the significance of genetic variations that exist for GBM [4,5]. Some of the genetic variations identified thus far have implications for survival and response to therapy. For example, mutation of isocitrate dehydrogenase (*IDH*) gene is associated with longer survival and O6-methylguanine-DNA methyltransferase promotor (*MGMT*) methylation is associated with improved response to therapy with temozolomide [6,7,8]. *IDH* mutation in particular is believed to be a fundamental branching point in genomic profiling of GBM and is included in the updated 2016 WHO classification scheme [9]. Growing evidence suggests that *IDH* mutation is a genetic marker of secondary glioblastoma arising from lower-grade astrocytoma [10] and that its presence leads to global hypermethylation [11]. Future changes to the WHO criteria are expected to further stratify patients on the basis of genetic information [12].

Given the existence of genetic variations in GBM that confer survival benefits, the ability to distinguish GBM’s with different genetic composition by imaging, i.e., the radiogenomics of GBM, can have significant impact on the prognostication and therapy selection for GBM patients in a non-invasive manner [13,14,15]. Multiple studies have, for example, determined that GBMs with *IDH* mutation tend to have larger proportion of nonenhancing tumor, less infiltrative tumor margin and frontal lobe location [16,17,18]. Ultimately, identifying the genetic information is based on pathologic testing, but the ability to predict genetic mutations from imaging information has been studied widely. Ideally, tumor imaging features available prior to surgery or biopsy could be used to make initial management decisions, such as guiding the extent of management. For example, a surgeon may choose a more aggressive resection in a patient with a known poor prognosis based on imaging features.

Other studies have shown rarer mutations that are also associated with a survival benefit. For example, a recent transcriptome analysis of long-term GBM survivors showed that concurrent gain of chromosomes 19 and 20 (hereafter referred to as 19/20 co-gain) is a positive prognostic factor in GBMs that lack the *IDH* mutation [19]. While incompletely understood, this genetic alteration is thought to have impacts on tumor-promoting, microglial driven inflammatory processes in GBM. However, the imaging findings of these tumors on magnetic resonance imaging (MRI) have not been studied. The aim of this study was to see if GBMs with 19/20 co-gain exhibit imaging features that are distinct from GBMs without the chromosomal co-gain. While this is a relatively rare mutation, this study provides information about the prospects of using MRI to identify tumors that may potentially have mutations that alter patient prognosis as well as their interaction with more commonly studied mutation.

## 2. Materials and Methods

### 2.1. Patient Population

This retrospective study was approved by the Institutional Review Board. Procedures for data collection and analysis complied with the Health Insurance Portability and accountability Act and were performed under a formal exemption for informed consent.

Review of the electronic medical record at a single institution from 2014–2018 identified 21 patients who were diagnosed with *IDH*-wildtype, primary glioblastoma and found to have 19/20 co-gain on chromosomal microarray. We only included patients with *IDH*-wildtype because the survival benefit of 19/20 co-gain was reported specifically in the context of *IDH*-wildtype patients. Of them, 18 patients had known *MGMT* methylation status and preoperative MRI that included T1 pre- and post-contrast, FLAIR, and DWI sequences. Three patients were excluded because they had incomplete pre-operative imaging information.

In our institution, the diagnosis of GBM includes assessment utilizing immunohistochemistry for the *IDH1* R132H mutant protein (Clone H09, Dianova, Hamburg, Germany), mutation panel testing for less-common *IDH* mutations (Multiplex PCR, Integrated DNA Technologies, Coralville, IA, USA), chromosomal microarray (Oncoscan^®^ molecular inversion probe array, ThermoFisher, Waltham, MA, USA) for detection of DNA copy number aberrations, and *MGMT* promoter methylation analysis (PCR/MassARRAY/MALDI-TOF assay, ARUP Laboratories, Salt Lake City, UT, USA). These tests in combination allow for the detection of *IDH* mutations, *MGMT* promoter methylation, and copy number aberrations across the genome, including chromosomes 19 and 20. Genomic alterations found in these analyses are reported as a part of routine clinical diagnosis.

To identify control patients, a separate review of the electronic medical record from 2014–2015 was conducted for patients who were diagnosed with primary glioblastoma and underwent adjuvant chemoradiation therapy at our institution. Of them, 25 patients were *IDH*-wildtype, had known *MGMT* methylation status, and were known to not have chromosome 19/20 co-gain on chromosomal microarray analysis. Nineteen of these patients had preoperative MRI that included T1 pre- and post-contrast, FLAIR, and DWI sequences. Six patients were excluded because they did not have complete imaging data.

### 2.2. Image Analysis

**Figure 1:**
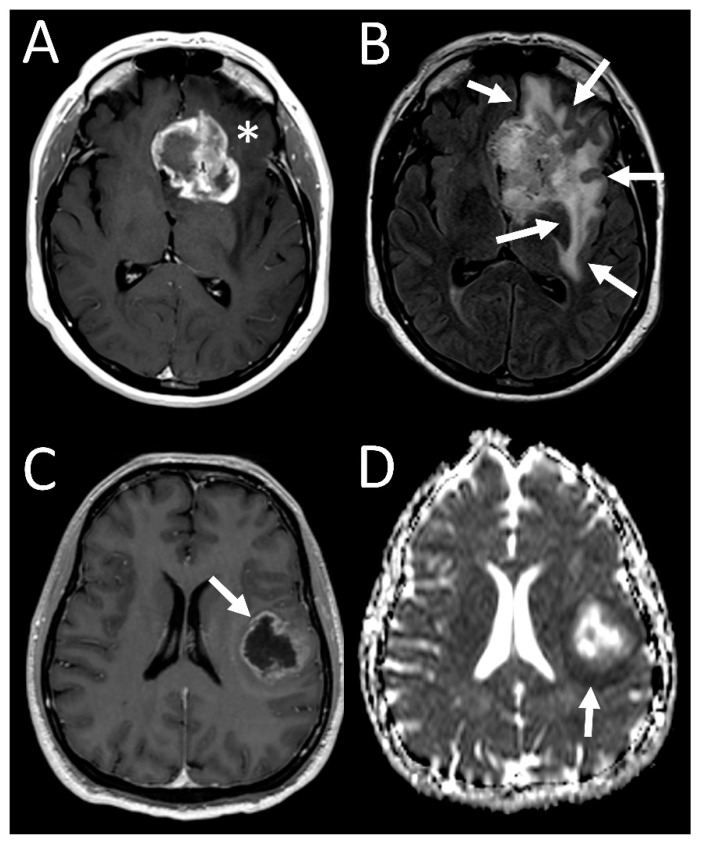
Sample imaging features of glioblastoma with 19/20 co-gain. (**A**) T1 postcontrast image of left frontal tumor demonstrating avid enhancement (asterisk), with (**B**) FLAIR image showing a large area of surrounding edema (arrows). (**C**) T1 postcontrast image of another tumor shows thinner rim of enhancing tumor (arrow), with (**D**) ADC map showing an area of non-enhancing, diffusion-restricting tumor beyond the enhancing tumor (arrow).

Preoperative MRI exams were performed at 1.5- or 3.0 Tesla following routine clinical protocol. Images included diffusion-weighted imaging, axial FLAIR, sagittal and axial pre-contrast T1, axial T2 with fat saturation, axial T1 post-contrast, and sagittal 3-D post-contrast T1. The size of the enhancing tumor was manually identified as the longest distance measured on a contiguous region of T1 post-contrast enhancement, using a single axial slice T1 post-contrast image that showed the largest extent of such finding. Size of the non-enhancing tumor was manually identified as the longest distance measured on a contiguous region of T1 hypointensity with perceived mass effect, using a single axial slice T1 pre-contrast image that showed the largest extent of such finding. The size of the edema surrounding the tumor was manually identified as the longest distance measured on a contiguous region of FLAIR hyperintensity with corresponding T1 hypointensity, with or without perceived mass effect, using a single axial slice FLAIR image that showed the largest extent of such finding. In all cases, edema was totally inclusive of non-enhancing tumor, which in turn was totally inclusive of the enhancing tumor (Figure 1).

Location of the enhancing tumor was classified as being centered in frontal, parietal, occipital or temporal lobe, and by laterality. Extension of the tumor or the surrounding edema into the corpus callosum or the basal ganglia was noted. Presence or absence of additional tumor features were noted: hemorrhage, ependymal extension, multifocality/multicentricity, satellite lesions, diffusion restriction, and pial invasion. A detailed description of these imaging features can be found in in the VASARI lexicon [20]. All image analysis was performed manually made using tools on the clinical PACS system (Centricity, GE, Milwaukee, Brookfield, WI, USA).

### 2.3. Statistical Analysis

Statistical analysis was performed using the Matlab software (R2018a, Mathworks, Portola Valley, CA, USA). Average values were calculated as the mean. When shown, error ranges represent the standard error of the mean. Classifications with two categories were compared using two-tailed Fisher’s exact test. Classifications with more than two categories were compared using Chi-square test. Continuous variables were compared using unpaired *t*-test. Values were compared directly for both groups as well as adjusting for *MGMT* status.

## 3. Results

### 3.1. Patient Population

A total of 18 patients with 19/20 co-gain and 19 patients without 19/20 co-gain met the selection criteria. The two cohorts demonstrated similar age and gender distribution, as well as similar rates of *MGMT* methylation status (Table 1).

### 3.2. Tumor Location 

In the control group, *MGMT* methylated tumors tended to be in the left hemisphere and *MGMT* unmethylated tumors in the right hemisphere (*p* = 0.0023, Fisher’s exact test). No such *MGMT* methylation-dependent laterality preference was seen in the 19/20 co-gain group (Table 2). Without the *MGMT* methylation status subclassification, tumors were equally likely to be in either cerebral hemisphere in both groups.

Distribution of the tumor among the four lobes of the supratentorial brain did not deviate significantly from equal distribution in either group (*p* > 0.05, Chi-square test). It also did not differ significantly between the two groups (*p* > 0.05, Chi-square test). In each group, two patients had tumors extending to the corpus callosum.

### 3.3. Tumor Size

*MGMT* methylated tumors were significantly smaller than *MGMT* unmethylated tumors in the control group (*p* = 0.0065, *t*-test). No such *MGMT* methylation-dependent tumor size difference was seen in the 19/20 co-gain group (Table 3). Average tumor size was similar between the two groups. Size of edema was not significantly different between any of the analyzed groups.

### 3.4. Tumor Characteristics

Pial invasion was seen more frequently in the control group (Table 4), reaching statistical significance (*p* = 0.05, Fisher’s exact test). None of the other VASARI features demonstrated significant difference between the two groups.

**Figure 2:**
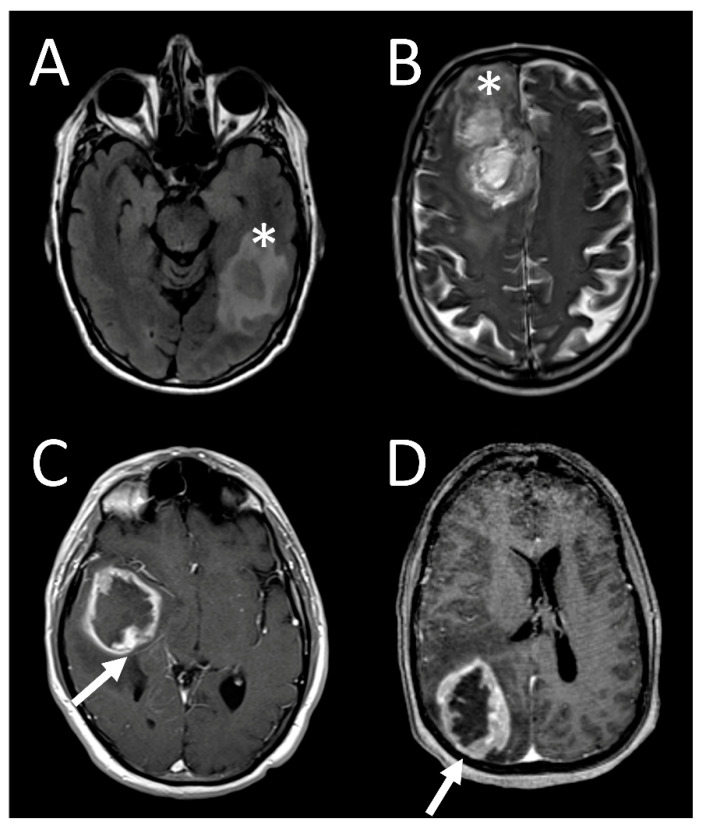
Sample imaging features of glioblastoma without 19/20 co-gain. FLAIR image of *MGMT* methylated tumor located in the left temporal lobe (**A**, asterisk). T2 images of *MGMT* unmethylated tumor located in the right frontal lobe (**B**, asterisk). T1 postcontrast image of *MGMT* unmethylated tumor located in the right temporal lobe demonstrating ependymal extension (**C**, arrow). T1 postcontrast image of *MGMT* unmethylated tumor located in the right parietal lobe demonstrating pial invasion posteriorly (**D**, arrow).

*MGMT* methylated tumors were less likely to demonstrate ependymal extension than *MGMT* unmethylated tumors in the control group (Figure 2), reaching statistical significance (*p* = 0.01, Fisher’s exact test). No *MGMT* methylation-dependent difference in the rate of ependymal extension was seen in the 19/20 co-gain group (Table 5). None of the other VASARI features demonstrated significant *MGMT* methylation-dependent difference (Table S1).

Regardless of the presence of 19/20 co-gain, all tumors demonstrated marked enhancement with well-defined margin of enhancement. Cyst formation was seen in only one of the 37 tumors examined in this study (a 19/20 co-gain tumor).

### 3.5. Overall Survival

Patients in the control group showed overall survival range of 3 to 69 months, with mean of 14.1 months. Patients in the 19/20 co-gain group showed overall survival range of 3 to 83 months, with mean of 18.6 months. The two groups did not reach statistically significant difference (*p* = 0.44, *t*-test). Within the control group, *MGMT* methylated patients showed longer overall survival (average 23.7 vs. 8.5 months, *p* = 0.05, *t*-test). No *MGMT* methylation-dependent difference in overall survival was seen in the 19/20 co-gain group (Supplemental Table S1).

## 4. Discussion

Besides the destructive and infiltrative biological behavior often manifested on imaging studies, glioblastoma’s marked genetic heterogeneity is believed to be a major contributing factor to its relative therapeutic resistance [21,22]. To this end, recent efforts utilizing next-generation sequencing (NGS) have identified several distinct molecular GBM subtypes of clinical significance, classified by genetic and epigenetic changes [5,23,24]. This led to the WHO including molecular subtypes of GBM in their latest central nervous system tumor classification [9]. *IDH* mutation and *MGMT* methylation are both considered positive prognostic factors and their molecular characterization is now standard of care in pathologic diagnosis. Analysis of other key genes implicated by the sequencing studies, including *EGFR*, *ATRX*, *CDK4*, *CDKN2A/B*, and *TERT*, may also become part of the routine molecular pathologic analysis [12]. These genetic and molecular features may be important for making initial management decisions as well as tracking changes in the tumor which may occur in recurrent glioblastoma that can guide changes in therapy [25,26].

With genomics of GBM increasingly becoming part of pathologic diagnosis and prognostication, there have been growing parallel efforts to correlate imaging findings to the genomic variations of clinical importance. Previous reports show that GBMs with *IDH* mutation tend to be located in the frontal lobes with larger proportion of nonenhancing tumor, smaller area of necrosis, and diminished blood flow on perfusion imaging [16,18,27,28,29]. GBMs with *MGMT* methylation on the other hand demonstrate left temporal lobe location and less diffusion restriction in addition to a larger proportion of the nonenhancing tumor [16,18,30,31].

These results are corroborated in our study. In our control group without the 19/20 co-gain, *MGMT* methylated tumors showed statistically significant preference for the left hemisphere. None of the GBMs analyzed in our study had the *IDH* mutation and no frontal lobar preference was observed, in line with previous studies. Interestingly, concurrent gain of chromosomes 19 and 20 interfered with localization preference—*MGMT* methylated tumors with 19/20 co-gain were just as likely to be found in the right hemisphere as left hemisphere. Further, 19/20 co-gain also nullified the *MGMT* unmethylated tumor’s tendency to demonstrate pial invasion. Pial invasion is a feature of aggressive tumors, and absence in the 19/20 co-gain group may be one of the reasons for better reported survival.

Previous studies have found that preoperative tumor size is not a significant prognostic factor [32,33,34], whereas the volume of postoperative residual tumor does affect survival. Tumor size is also not significantly featured in imaging studies of GBM possessing different genetic subtypes. Interestingly, our control group demonstrated significant tumor size difference depending on *MGMT* methylation, with *MGMT* methylated tumors demonstrating smaller size. The reason for this difference is uncertain, although MGMT unmethylated tumors may have aggressive features that lead to larger tumor size. In the 19/20 co-gain group, however, there was no significant size difference depending on *MGMT* methylation status. This finding, similar to 19/20 co-gain nullifying laterality preference of *MGMT* methylation tumors, supports the hypothesis that tumors possessing 19/20 co-gain behave in a different biological manner than those that do not possess the chromosomal gains in a way that is independent of *MGMT* methylation status or *IDH* mutation.

Our study was limited by the fact that it was a small, single-institutional study. Although isolated gains of chromosomes 19 or 20 is common in GBM [24], there is only anecdotal evidence of their co-occurrence [19,35]. Additionally, although we controlled for the potential confounding factor of *IDH* mutation by only including patients with wildtype *IDH*, there could be other confounding genetic factors that could not be controlled due to the small sample size. Despite these limitations, it is the first study, to our knowledge, to investigate the imaging features of a unique genetic variation in GBM that has been demonstrated to be a clinically significant positive prognostic factor independent of *IDH* mutation. Further, our results of 19/20 co-gain nullifying, or interfering with previously observed imaging trends of *MGMT* methylation, suggest that imaging prognostication based on case-by-case correlation with single genetic alteration may not be a practical approach. Instead, the more practical approach may be to approach imaging prognostication in a more holistic manner.

Our study, as well as many previous studies, find that imaging features that are generally considered to be signs of aggressive disease, such as contrast enhancement, diffusion restriction, and invasion of adjacent structures, are correlated with genetic alterations that confer worse prognosis. On the other hand, benign imaging features such as nonenhancement and cyst formation are correlated with genetic alterations with improved prognosis. The prognostic significance of topology, i.e., the location of the tumor, is less intuitive, and the fact that certain locations such as frontal lobe and left temporal lobe are seen with genetic alterations of better prognosis may be related to the tumor’s biological behavior, or the fact that certain locations are more easily approached surgically and that post-surgical functional losses are not as profound.

## 5. Conclusions

In this paper, we have assessed the imaging findings of GBM patients with a rare mutation, 19/20 co-gain, which has been associated with a better prognosis. These tumors had similar imaging features compared to GBMs without the mutation when considered alone, although when considering together with MGMT methylation, differences were apparent. Our results indicate that different combinations of genetic and molecular features of GBM, including *IDH* mutation and *MGMT* methylation, may have unpredictable effects on imaging appearance. A suggested future direction is to analyze a large population of glioblastoma and extract several key component imaging features that are correlated with improved patient outcome, potentially with help of machine learning algorithms. This type of approach considering a global genomic profile may be superior to assessing individual genetic alterations and can potentially better assess imaging features of individual mutations and their interactions which are not yet incompletely understood. Such a system can then be used in a clinical setting to provide physicians valuable prognostic information they can use to make decisions about clinical management.

## Figures and Tables

**Table 1:** Patient characteristics.

	Control Group	19/20 Co-Gain Group
Total patients	19	18
Average age in years	61.8 ± 2.4	61.7 ± 2.2
Male/female	13/6	12/6
*IDH*	Wildtype	Wildtype
*MGMT* methylated/unmethylated	7/12	7/11

**Table 2:** Tumor location.

Control Group		Frontal	Parietal	Occipital	Temporal	Sum
*MGMT* methylated	Right	0	0	0	0	0
	Left	0	1	2	4	7
*MGMT* unmethylated	Right	2	3	2	2	9
	Left	0	0	1	1	2
Sum		2	4	5	7	18 *
19/20 co-gain group		Frontal	Parietal	Occipital	Temporal	Sum
*MGMT* methylated	Right	1	2	1	0	4
	Left	2	0	0	1	3
*MGMT* unmethylated	Right	1	0	3	1	5
	Left	1	3	0	2	6
Sum		5	5	4	4	18

* One patient in the control group had tumor in the right basal ganglia and was excluded from this table.

**Table 3:** Tumor size. Mean maximum of tumor edema and enhancing region (ENH) in cm. Values are shown for MGMT methylated (MGMT+) and unmethylated (MGMT−).

	Control Group			19/20 Co-Gain Group			*p*-Value (*t*-Test)
Edema	7.3 ± 0.5			7.1 ± 0.5			0.84
ENH tumor	4.7 ± 0.3			4.5 ± 0.3			0.64
	*MGMT+*	*MGMT*−	*p*-value (*t*-test)	*MGMT*+	*MGMT*−	*p*-value (*t*-test)	
Edema	6.5 ± 1.0	7.7 ± 0.4	0.21	7.4 ± 1.4	7.0 ± 0.6	0.72	
ENH tumor	3.6 ± 0.3	5.4 +/− 0.4	0.0065	4.4 ± 0.7	4.5 ± 0.5	0.92	

**Table 4:** Tumor characteristics.

	Control Group	19/20 Co-Gain Group	*p*-Value (Fisher’s Exact Test)
T1 to FLAIR ratio (expansive/mixed or infiltrative)	4/15	4/14	1.00
Hemorrhage (present/absent)	12/7	11/7	1.00
Ependymal extension (present/absent)	13/6	11/7	0.74
Multifocal or multicentric (yes/no)	5/14	6/12	0.73
Satellites (present/absent)	7/12	5/13	0.73
Diffusion restriction (present/absent)	12/7	9/9	0.51
Pial invasion (present/absent)	13/6	6/12	0.05

**Table 5:** Ependymal extension.

	Control Group		19/20 Co-Gain Group	
	*MGMT* Methylated	*MGMT* Unmethylated	*MGMT* Methylated	*MGMT* Unmethylated
Present	2	11	5	6
Absent	5	1	2	5
*p*-value (Fisher’s exact test)	0.01		0.64	

## Data Availability

The data presented in this study are available on request from the corresponding author. The data are not publicly available due to privacy concerns for sharing patient data.

## Supplementary Materials

The material is available online at https://www.mdpi.com/article/10.3390/tomography7020021/s1.
