# Supplementary material for: MRI Imaging Characteristics of Glioblastoma with Concurrent Gain of Chromosomes 19 and 20
Source: Tomography. 2021 Jun 2;7(2):228–37. doi: 10.3390/tomography7020021 (PMC8293438; doi:10.3390/tomography7020021)
Supplement: Supplementary file 1 [file tomography-07-00021-s001.zip › tomography-1205197-supplementary.pdf]

	MGMT metylation	T1/Flair ratio (expansive = 0)	Diffusion restriction	Pial invasion	Ependymal extension	Hemorrhage	Multifocal or multicentric	Satellites	Overall survivial (months)
MUT001	0	0	1	0	0	1	0	1	18
MUT002	0	0	0	1	1	1	1	0	20
MUT003	0	1	0	0	0	0	0	0	15
MUT004	0	1	1	0	1	1	1	0	22
MUT005	0	1	1	1	1	1	0	0	5
MUT006	0	1	0	0	1	1	0	0	31
MUT007	0	1	1	1	0	1	0	0	13
MUT008	0	1	0	0	1	0	0	1	4
MUT009	0	1	0	0	0	1	0	0	31
MUT010	0	1	0	0	0	0	1	1	8
MUT011	0	1	1	0	1	1	0	0	8
MUT012	1	1	1	0	1	1	0	0	83
MUT013	1	0	1	1	1	1	1	0	30
MUT014	1	1	1	1	1	1	0	0	15
MUT015	1	1	0	0	1	0	1	1	6
MUT016	1	0	1	0	0	0	1	1	7
MUT017	1	1	0	0	1	0	0	0	16
MUT018	1	1	0	1	0	0	0	0	3
p-value comparing the met+ / met- groups (Fisher's two tail exact, t-test)		1.00	1.00	0.63	0.64	0.33	0.63	1.00	0.45
CON001	0	0	1	1	1	1	0	0	16
CON002	0	1	0	1	1	0	0	0	5
CON003	0	1	0	0	1	1	0	0	13
CON004	0	1	1	0	0	1	0	0	5
CON005	0	0	1	0	1	0	0	0	9
CON006	0	1	1	1	1	1	0	0	2
CON007	0	1	1	1	1	1	1	1	9
CON008	0	1	0	1	1	1	0	1	9
CON009	0	1	1	1	1	1	0	0	7
CON010	0	0	1	1	1	1	0	0	6
CON011	0	1	1	1	1	1	1	0	10
CON012	0	1	0	1	1	0	0	1	11
CON013	1	1	1	1	0	1	0	0	19
CON014	1	1	0	0	1	0	1	1	7
CON016	1	1	1	1	1	1	0	1	9
CON017	1	1	0	0	0	0	0	1	49
CON018	1	0	1	1	0	0	0	0	69
CON019	1	1	1	1	0	0	1	0	10
CON020	1	1	0	0	0	1	1	1	3
p-value comparing the met+ / met- groups (Fisher's two tail exact, t-test)		1.00	1.00	0.62	**0.01**	0.33	0.30	0.33	**0.05**

p-value comparing the overall survival between the control and the 19/20 co-gain groups = 0.44 (t-test)
